# The Ocular Conjunctiva as a Mucosal Immunization Route: A Profile of the Immune Response to the Model Antigen Tetanus Toxoid

**DOI:** 10.1371/journal.pone.0060682

**Published:** 2013-04-26

**Authors:** Talin Barisani-Asenbauer, Aleksandra Inic-Kanada, Sandra Belij, Emilija Marinkovic, Ivana Stojicevic, Jacqueline Montanaro, Elisabeth Stein, Nora Bintner, Marijana Stojanovic

**Affiliations:** 1 OCUVAC – Center of Ocular Inflammation and Infection, Laura Bassi Centers of Expertise, Institute of Specific Prophylaxis and Tropical Medicine, Center for Pathophysiology, Infectiology and Immunology, Medical University of Vienna, Vienna, Austria; 2 Department of Research and Development, Institute of Virology, Vaccines and Sera – TORLAK, Belgrade, Serbia; Instituto Butantan, Brazil

## Abstract

**Background:**

In a quest for a needle-free vaccine administration strategy, we evaluated the ocular conjunctiva as an alternative mucosal immunization route by profiling and comparing the local and systemic immune responses to the subcutaneous or conjunctival administration of tetanus toxoid (TTd), a model antigen.

**Materials and methods:**

BALB/c and C57BL/6 mice were immunized either subcutaneously with TTd alone or via the conjunctiva with TTd alone, TTd mixed with 2% glycerol or TTd with merthiolate-inactivated whole-cell *B. pertussis* (wBP) as adjuvants. Mice were immunized on days 0, 7 and 14 via both routes, and an evaluation of the local and systemic immune responses was performed two weeks after the last immunization. Four weeks after the last immunization, the mice were challenged with a lethal dose (2 × LD_50_) of tetanus toxin.

**Results:**

The conjunctival application of TTd in BALB/c mice induced TTd-specific secretory IgA production and skewed the TTd-specific immune response toward a Th1/Th17 profile, as determined by the stimulation of IFNγ and IL-17A secretion and/or the concurrent pronounced reduction of IL-4 secretion, irrespective of the adjuvant. In conjunctivaly immunized C57BL/6 mice, only TTd administered with wBP promoted the establishment of a mixed Th1/Th17 TTd-specific immune response, whereas TTd alone or TTd in conjunction with glycerol initiated a dominant Th1 response against TTd. Immunization via the conjunctiva with TTd plus wBP adjuvant resulted in a 33% survival rate of challenged mice compared to a 0% survival rate in non-immunized animals (p<0.05).

**Conclusion:**

Conjunctival immunization with TTd alone or with various adjuvants induced TTd-specific local and systemic immune responses, predominantly of the Th1 type. The strongest immune responses developed in mice that received TTd together with wBP, which implies that this alternative route might tailor the immune response to fight intracellular bacteria or viruses more effectively.

## Introduction

The conjunctiva, which lines the ocular surface from the corneal rim to the lid margin [1], and its underlying structures are now recognized as a part of the mucosa-associated lymphoid tissue (MALT), annotated as conjunctiva-associated lymphoid tissue (CALT) or eye-associated lymphoid tissue (EALT) [2]. CALT has the typical components of a physiologically protective mucosal immune system, as it contains diffuse lymphoid tissues and lymphoid follicles that form the efferent and afferent limbs, respectively, of lymphoid tissue. Thus, CALT can detect antigens from the ocular surface, present the antigens and generate protective effector cells; together, these properties signify the presence of a mucosal immune system at the conjunctiva [3], [4], [5], [6]. Theoretically, the conjunctiva is an attractive choice for mucosal immunization, particularly against ocular infections, as eye drops are easily administered; drop-count dosing is feasible; and as the conjunctiva is interconnected with the nasal mucosa via the tear ducts, the administration of antigens to the conjunctival sac would additionally drain to the nasal-associated lymphoid tissue (NALT). Nevertheless, the conjunctival route has been exploited predominately only in veterinary applications in the use of live attenuated vaccines, which have proven to be efficient against various infectious diseases [7], [8]. Several vaccines that use this strategy have been licensed, including poultry vaccines against *Newcastle virus*, infectious bursitis virus, chicken herpes virus and turkey herpes virus; feline vaccines against viral rhinotracheitis, calicivirus and panleukopenia; and goat and sheep vaccines against *Brucella melitensis* [9], [10], [11], [12], [13].

For human use, live attenuated vaccines have certain limitations: there is a possibility that an attenuated microbe in a vaccine could revert to a virulent form and cause disease, subjects who have damaged or weakened immune systems cannot safely receive the vaccines, and the vaccines often require refrigeration to remain viable [14]. In contrast, subunit vaccines, which are based on purified protective proteins or carbohydrates, might be a safe alternative to live attenuated vaccines [15]. However, little is known about the conjunctival immunization and its immune response profile concerning protein subunit vaccines, particularly compared to systemic vaccination. Previous comparative studies on conjunctival immunization have been performed, albeit with certain constraints: 1) two different effects were compared, e.g., immune responses elicited with a live attenuated vaccine administered subcutaneously were compared to responses elicited with a subunit vaccine applied via the conjunctiva [16]; 2) immune responses after conjunctival immunization with either live attenuated vaccines or peptides were assessed without comparison to systemic routes [17], [18], [19]; 3) in lieu of an immune response, anergy was induced following the administration of different doses of ovalbumin twice per day for 10 days via the conjunctiva following the subcutaneous administration of Ag [20]. Consequently, an immune response profile of the mucosal/conjunctival route versus the parenteral/subcutaneous route of immunization using protein subunits in combination with suitable ocular adjuvants is currently absent. Therefore, the aim of our study is first to undertake a comparative study of the local and systemic immune responses after conjunctival and subcutaneous immunizations using TTd as a model antigen. The overall rationale for choosing TTd was that this antigen has previously been used as a valuable model antigen [21], and TTd is well known as a strong immunogen [22]. Furthermore, as TTd is an active component of the vaccine against tetanus, the immune response obtained via subcutaneous immunization with TTd is well characterized and can be used as a “gold standard.”

Second, because many ocular surface pathogens are either intracellular bacteria or viruses that require a (Th1) for clearance, we further assessed the influence of Th1-promoting adjuvants, applied together with the antigen, on the immune response induced by conjunctival immunization while ensuring that the choice of adjuvant was tolerated by the ocular surface.

Third, we used two genetically different inbred mouse strains, BALB/c and C57BL/6 (with predominantly Th2 and Th1 immune responses, respectively), to gain a broader understanding of the immune response elicited by conjunctival immunization.

## Materials and Methods

### Mice

Eight-week-old BALB/c and C57BL/6 female mice were used in the experiments. All experiments were approved by the “Ethics Committee for the Welfare of Experimental Animals” and by the committee section at the Institute of Virology, Vaccines and Sera – Torlak and conformed to the Serbian laws and European regulations on animal welfare (Approval No. 011-00-00510/2011-05/2).

### Antigens, Adjuvants and Immunization and Bleeding Schedules

BALB/c and C57BL/6 female mice were immunized via the conjunctiva (conj//) with TTd (Institute of Virology, Vaccines and Sera – Torlak, Belgrade, Serbia) as a model antigen (100 µg TTd/PBS per mouse in 10 µl (5 µl per eye) was applied to the conjunctiva), and 2% glycerol (glyc) and merthiolat-inactivated whole cell *B. pertussis* (wBP) (Institute of Virology, Vaccines and Sera – Torlak, Belgrade, Serbia) were used as Th1-promoting adjuvants. These adjuvants were chosen because glyc is commonly used in eye drops for human use at this concentration and because preliminary experiments in which wBP was administered in a corpusculated form or as an adjuvant platform revealed no visible signs of inflammation or infection at the ocular surface. Mice (n = 10 per group) were immunized on days 0, 7 and 14 via either the conj//or sc//routes, and the evaluations of local and systemic immune responses were conducted two weeks after the last immunization. According to the applied immunization protocols, the mice were divided into the following four experimental groups: conj//TTd, conj//TTd/glyc, conj//TTd/wBP and sc//TTd. Age-matched, non-immunized mice and conj//glyc- and conj//wBP-treated mice were used as controls.

All vaccines were prepared *in situ* by mixing defined volumes of the stock TTd and wBPsolutions (both in PBS) or concentrated glycerol (stock solution 85%) in order to prepare vaccines that finally contain:

- 10 mg/ml TTd in PBS for conj//TTd
- 10 mg/ml TTd and 2% glycerol in PBS for conj//TTd/glyc
- 10 mg/ml TTd and 2×10^8^ cells/ml wBP in PBS for conj//TTd/wBP
- 1 mg/ml TTd in PBS for sc//TTd
- 2% glycerol in PBS for conj//glyc
- 2×10^8^ cells/ml wBP in PBS for conj//wBP

Obtained solutions were mixed thoroughly by vortexing for 30 minutes and 5 µl per eye (in total 10 µl per mice) were applied via conjunctiva. 100 µl per mouse was applied subcutaneously.

Mice that were immunized via the conjunctiva were anaesthetized by intraperitoneal (i.p.) administration of a mixture of xylazine (Sigma-Aldrich, Kansas, KS, USA) and ketamine (Richter Pharma AG, Wels, Austria). Antigens were applied onto the conjunctival sacs using a micropipette. The mice were maintained under anesthesia for 30 minutes to prevent removal of the immunization solution.

Subcutaneously immunized (sc//) mice were used as a “gold standard” (100 µg TTd/PBS per mouse in 100 µl). The TTd used for the immunizations met the standards for specific and reversed toxicity according to the European Pharmacopoeia requirements.

### Sample Collection

Samples of blood sera were collected by bleeding from the retro-orbital sinuses two weeks after the completion of the immunization protocol. The collected sera were complement depleted, aliquoted and stored at –20°C. Tear-wash samples were obtained by lavage with 10 µl PBS per eye. The collected tears were supplemented with a protease inhibitor cocktail (Thermo Scientific, USA) and stored at –80°C.

### Detection of TTd-specific IgG, IgG Subclasses and IgA in Mouse Sera and Tears

ELISA plates (MaxiSorp; Nunc, Roskilde, Denmark) were coated (50 µl/well) with TTd (2.5 µg/ml TTd in PBS) by overnight adsorption at 4°C, and 1% (w/v) BSA/PBS was applied as a blocking reagent for 2 h at room temperature. This blocking step, as well as all subsequent ELISA steps, was followed by a wash with 0.05% (v/v) Tween 20 in PBS (four times, 200 µl/well). Appropriately diluted non-pooled serum (1∶100) and tear (1∶2) samples were incubated in the plates for 1 h at room temperature. Ag-specific antibody binding was detected with after 1 h incubation at room temperature using biotin-labeled anti-mouse IgG (Sigma, Steinheim, Germany), biotin-labeled anti-mouse IgG1, IgG2a, and IgG2c antibodies (Sigma, Steinheim, Germany) and biotin-labeled anti-mouse IgA antibody (BioLegend). Antigen-antibody interactions were visualized using the extrAvidin-peroxidase/o-phenylendiamine system (Sigma, Steinheim, Germany), and absorbance was recorded at 492/620 nm (A_492/620_). The cutoff value for each system was defined according to the A_492/620_ value obtained from “negative control” wells (1% BSA w/v in PBS as a sample) plus 3×SD. Samples were considered positive when the A_492/620_ value exceeded the cutoff value.

### Cells from Draining Lymph Nodes

Submandibular (SMLN) lymph nodes from mice that were immunized via the conjunctiva and from control mice were aseptically isolated, trimmed of all excess tissue and placed in sterile complete RPMI 1640 (Sigma-Aldrich) supplemented with inactivated 5% fetal calf serum (FCS). Lymphocytes were harvested in 5% FCS/RPMI 1640 and passed through sterile steel mesh to remove large particles. Cell suspensions were centrifuged at 1000 rpm (SIGMA 3K18, Sigma Laboratory Centrifuges GmbH) to yield pellets. After centrifugation, the lymphocytes were washed three times in 5% FCS/RPMI 1640 with centrifugation at 1000 rpm (5 min). The cells were finally diluted in 10% FCS/50 µM β-mercaptoethanol/RPMI 1640 to a concentration of 2×10^6^ cells/ml. The viability of these cell preparations, as determined by trypan blue exclusion, was greater than 95%. Lymphocytes (2×10^6^ cells/ml) were used for ELISA or were stimulated *in vitro* (37°C, 5% CO_2_, 48 h). As a stimulus, TTd was added to the cultures at 5 µg/ml.

### Abundance of TTd-specific B cells within the Total mIgG^+^ B cell Population in Draining Lymph Nodes

The abundance of the specific B cell population was investigated using an ELISA-based procedure. SMLN suspensions were simultaneously assessed for both the amount of total mIgG^+^ cells as well as TTd-specific mIgG^+^ B cells. All washes were conducted in PBS. For the detection of total mIgG^+^ B cells, MaxiSorp microtiter plates (Nunc) were coated (50 µl/well, overnight at 4°C) with commercially available polyclonal anti-mouse IgG (10 µg/ml PBS; Sigma M 0659). Suspensions containing 2×10^5^ cells/ml were added to the blocked (1% BSA in PBS) anti-mouse IgG-coated wells (50 µl/well) to assess the total number of mIgG^+^ B cells. The coating procedure that was previously described in the Materials and Methods section (for the detection of IgG and IgA specific for TTd in the sera and tears) was applied for the detection of TTd-specific B cells. For this assessment, suspensions containing 2×10^6^ cells/ml were used (50 µl/well). TTd-specific and total mIgG^+^ B cells were detected using polyclonal alkaline phosphatase-labeled anti-mouse IgG (Sigma, A-3562). Non-specific binding was assessed in wells that were blocked but were not coated with reagents (i.e., those lacking capture antibody and specific antigen). The amount of bound alkaline phosphatase-labeled antibodies per well was evaluated using p-nitrophenyl phosphate as a substrate. The transformation of p-nitrophenyl phosphate by alkaline phosphate occurred at the same rate in all wells. The abundance of specific B cells within the mIgG^+^ population was calculated for each individual animal. Using the A_405_ value measured upon the evaluation of total mIgG-expressing cells (A_405tot_) and the corresponding A_405_ value measured upon specific B cell assessment (A_405spec_), the relative abundance (RA) of specific B cells was calculated as follows: RA = (A_405spec_×100)/A_405tot_.

### MTT Assay

Following 48 h of incubation (5% CO2, 37°C), the plates containing the cell cultures were centrifuged (800 rpm, 10 min), and the supernatants were decanted. DMEM/25 mM HEPES/0.2% NaHCO_3_ containing 500 µg/ml of 3-(4,5-dimethylthiazol-2-yl)-2,5-diphenyltetrazolium bromide (MTT) (Sigma-Aldrich) was added to the experimental wells (100 µl/well), and the cells were incubated (37°C, 5% CO_2_, 4 h). Reactions were halted by the addition of 10% SDS/10 mM HCl (100 µl/well). After an overnight incubation at 37°C, absorbance values were measured at 580 nm (A_580_) using a spectrophotometer (Ascent 6–384 [Suomi], MTX Lab Systems Inc., Vienna, VA, USA). The number of viable cells per well (NVC) was calculated from a standard curve drawn as the number of cells plotted against A_580_. Standard curves were drawn for both mouse strains (BALB/c and C57BL/6). Discrete pools of non-stimulated cells were taken from each strain and, after counting the cells using a hemocytometer, were then used as standards. Standard suspensions were plated in serial dilutions prior to centrifugation and were then were treated identically to the experimental wells that received stimulated aliquots. A proliferation index (PI) for each stimulated cell suspension was calculated per individual source animal. The PI index was defined as the ratio of NVC present in stimulated (S) samples to NVC present in non-stimulated (nS) samples, such that PI = NVCS : NVCnS.

### Cytokine Profile of Draining Lymph Node Cells

The production of IFNγ, IL-4, IL-17A and IL-10 was analyzed by measuring the supernatant concentrations of non-stimulated and TTd-stimulated SMLN lymph node cultures using sandwich ELISAs with commercially available monoclonal antibodies (eBioscience). Unlabeled monoclonal Abs specific for IFNγ (1 µg/ml), IL-4 (2 µg/ml), IL-17A (1 µg/ml) or IL-10 (1 µg/ml) were coated onto microtiter plates (MaxiSorp, Nunc) by overnight adsorption at 4°C, and 1% BSA/PBS (w/v) was used to block the plates (2 h) at room temperature. Blocking and all subsequent ELISA steps were followed by a wash step with 0.05% (v/v) Tween 20 in PBS (four times, 200 µl/well). Lymph node supernatants were incubated in the plates (1 h) at room temperature. Biotin-labeled Abs specific for IFNγ (2 µg/ml), IL-4 (1 µg/ml), IL-17A (1 µg/ml) or IL-10 (1 µg/ml) and formulated in 1% BSA/PBS (w/v) were then added to the wells and incubated for 1 h at room temperature. The extrAvidin-alkaline phosphatase/p-nitrophenyl phosphate system (Sigma-Aldrich) was used to visualize antigen–Ab interactions. Absorbance was monitored at 405 nm (A_405_). The cutoff value for each system was calculated as the displayed A_405_ reading measured in the negative control well (1% BSA/PBS used as a sample) plus 3×SD. Standard curves were created using commercially available recombinant mouse IFNγ, IL-4, IL-17A and IL-10.

### Protection Assay Against Tetanus Toxin (TTn)

Four weeks after the course of conj//and sc//immunizations, anesthetized mice (n = 6) were challenged with a lethal dose (2×LD_50_) of TTn by intraperitoneal (i.p.) injection. The animals were monitored for weight loss and survival every day for 5 days.

### Tolerability at the Ocular Surface

Signs of ocular irritation were monitored in BALB/c and C57BL/6 mice immunized via the conjunctiva and in non-immunized controls daily during the course of immunization and every week for the remainder of the study by two unbiased observers. Conjunctival hyperemia, edema and corneal clarity were assessed using magnifying loupes.

### Statistical Analysis

The results are presented as the mean values ± standard error (SE). The statistical significance of the observed differences was evaluated using the *t*-test for independent groups. A probability (*P*) value of 0.05 was set as the limit of significance (software: ORIGIN 8.0). The correlation between variables was evaluated by Pearson’s bivariate correlation analysis (software: IBM SPSS Statistics 20).

## Results

### Secretory IgA (SIgA) Levels in Tears Increased in Both Mouse Strains Following TTd Immunization via the Conjunctiva versus Subcutaneous Immunization

**Figure 1:**
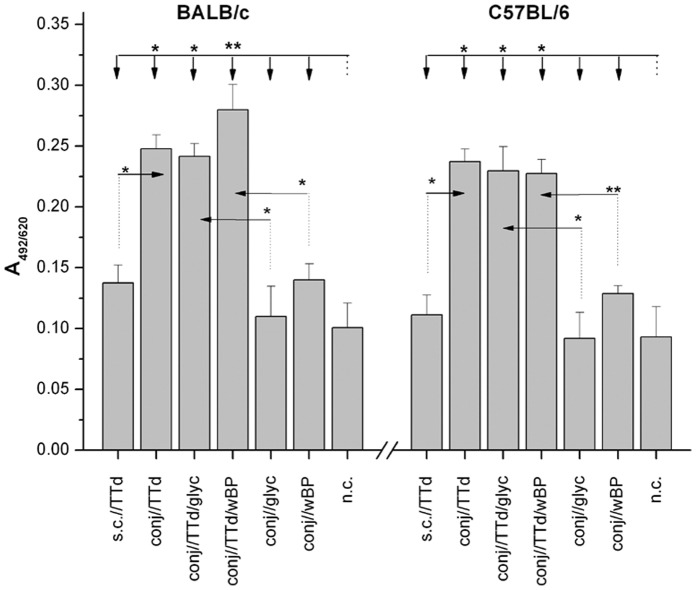
Levels of TTd-specific SIgA in tear washes from BALB/c and C57BL/6 mice that were immunized according to the assigned protocols. Samples were collected two weeks after the completion of the immunizations and were assayed by ELISA (dilution 1∶2). The results are presented as the mean A_492/620_± SE (n = 10). The significance of the observed differences was calculated by *t*-test (P<0.05*, P<0.005**). The reference group is indicated by a dotted line, and the comparison group is indicated by an arrow.

Immunization through the conjunctival route resulted in levels of TTd-specific SIgA in both mouse strains that were significantly higher than those found in the tears of syngeneic control mice (non-immunized and adjuvant-alone immunized) as well as mice that were subcutaneously immunized with TTd (Fig. 1). The amount of TTd-specific SIgA detected in the tears of sc//TTd-immunized mice was negligible and was similar to the levels found in tears from non-immunized mice. Although the mean levels of SIgA in the tears of conj//TTd/wBP-immunized mice were higher compared to levels in conj//TTd- and conj//TTd/glyc-immunized mice of the same strain, these differences were not significant. With regard to the different mouse strains, BALB/c mice exhibited greater SIgA concentrations in tears than C57BL/6 mice, although this difference was not significant.

### Serum IgG and IgA Levels Increased Following Conjunctival Immunization with TTd when wBP was used as an Adjuvant

**Figure 2:**
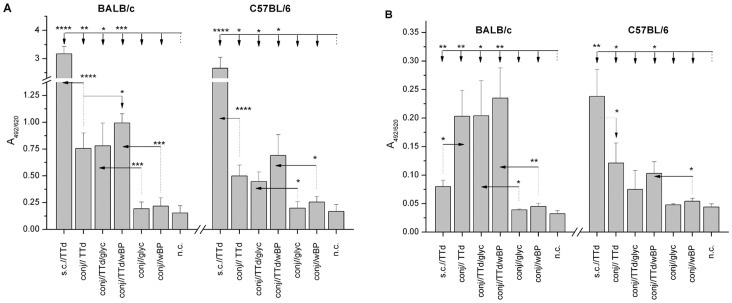
Levels of TTd-specific IgG (A) and IgA (B) in the sera of BALB/c and C57BL/6 mice immunized according to the assigned protocols. Serum samples were collected two weeks after the completion of the immunizations and were assayed by ELISA (dilution 1∶100). The results are presented as the mean A_492/620_± SE (n = 10). The significance of the observed differences was calculated by *t*-test (P<0.05*, P<0.005**, P<0.0005***, *P<0.00005*). The reference group is indicated by a dotted line, and the comparison group is indicated by an arrow.

In both mouse strains, conjunctival immunization with TTd, TTd/glyc or TTd/wBP resulted in significantly higher levels of TTd-specific IgG (Fig. 2a) and IgA (Fig. 2b) in the serum compared to serum from non-immunized or adjuvant-alone-immunized mice.

Generally, anti-TTd IgG and IgA levels in the serum of conjunctively immunized BALB/c mice were higher than the levels in the serum of C57BL/6 mice treated the same way. A comparison of the syngeneic mice immunized with TTd via the conjunctiva revealed that, except in the case of anti-TTdIgG in BALB/c mice, there were no significant differences in the levels of sera anti-TTd antibodies among the mice. The conjunctival administration of TTd mixed with wBP promoted a significantly higher (*P<0.05*) systemic production of TTd-specific IgG than the administration of TTd alone. In comparison to the “gold standard” (sc//immunization with TTd without adjuvant), the levels of serum TTd-specific IgG were significantly lower in all mice that were immunized with TTd via the conjunctiva (*P<0.0005* in comparison to sc//TTd-immunized mice of the same strain). Regarding the level of sera TTd-specific IgA, the conjunctival administration of TTd in BALB/c mice was superior to subcutaneous administration (*P<0.05*), whereas in C57BL/6 mice an opposite situation is recorded (*P<0.05*).

### Serum IgG Antibody Subclass Responses Provided the Initial Evidence that Immunization with TTd via the Conjunctiva can Induce Skewing of TTd-specific Immune Responses towards a Th1–type Response

**Figure 3:**
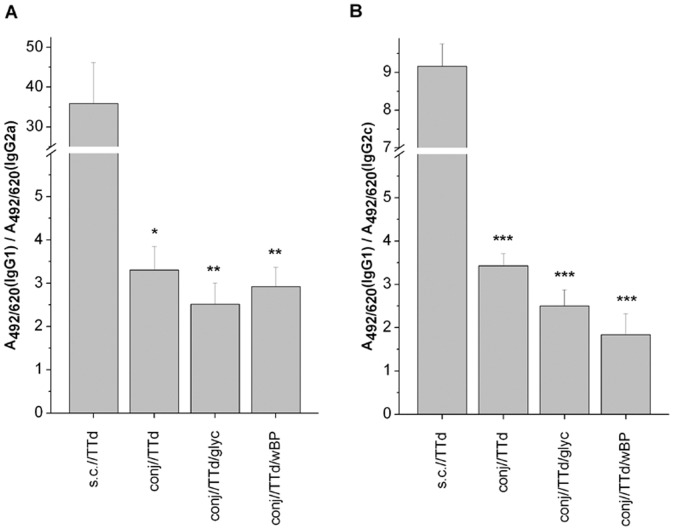
IgG1/IgG2a (A) and IgG1/IgG2c (B) ratios calculated for TTd-specific antibodies in the sera of TTd-immunized BALB/c and C57BL/6 mice, respectively. Serum samples were collected two weeks after the completion of the immunizations and were assayed by ELISA (dilution 1∶100). Ratios were determined using the A_405_ values obtained upon the measurement of TTd-specific IgG1 and IgG2a or IgG2c in sera. The results are presented as the mean A_405_(IgG1)/A_405_(IgG2a,c) ± SE (n = 10). The significance of the differences between syngeneic mice immunized subcutaneously with TTd (reference group) and those immunized via the conjunctiva was calculated by *t*-test (*P<*0.05*, *P<*0.005**, *P<*0.0005***).

When the TTd-specific serum IgG subclass immune responses were assessed (Fig. 3), the ratios of IgG1 and IgG2a in BALB/c mice and IgG1 and IgG2c in C57BL/6 mice were significantly higher in sc//−immunized mice in comparison to each group of mice that were immunized via the conjunctiva (*P<0.05* for BALB/c and *P*<0.0005 for C57BL/6). The rise in the abundance of IgG antibodies that are dependent on IFNγ for secretion (i.e., IgG2a in BALB/c and IgG2c in C57BL/6) in the TTd-specific sera IgG pool of conjunctively immunized mice indicated that Th1 immune response skewing had occurred. Although there were differences in the IgG1/IgG2a,c ratios among syngeneic conjunctively immunized mice, these differences were not significant.

### An Increased Population of TTd-specific B cells within the Total SMLN B cell Population Strongly Correlated with Levels of Sera TTd-specific Antibodies

**Figure 4:**
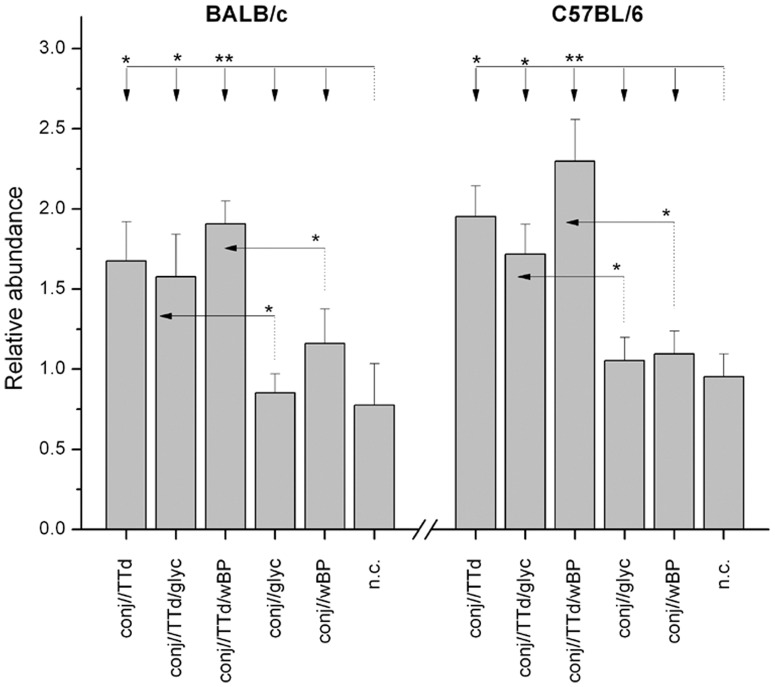
The relative abundance (RA) of TTd-specific mIgG^+^ B cells within the total population of mIgG^+^ B cells in SMLN upon completion of the assigned immunization protocols. The RA of the TTd-specific mIgG^+^ B cell population was calculated for each mouse. The results are presented as the mean RA ± SE for each experimental group of mice (n = 5). The statistical significance of the observed differences in TTd-specific mIgG^+^ B cell pool abundances was determined by *t*-test (P<0.05*, P<0.005**, P<0.0005***). The reference group is indicated by a dotted line, and an arrow indicates the comparison group.

An increased population of TTd-specific B cells was found within the total mIgG^+^ B cell population in the draining lymph nodes of mice immunized with TTd via the conjunctiva (Fig. 4). Compared to the syngeneic age-matched control group, as well as groups treated with glyc or wBP alone, a significantly higher frequency of mIgG^+^ TTd-specific B cells within the draining SMLN was evident in all these groups (*P<0.05*). The greatest abundance of TTd-specific B cells in mice immunized via the conjunctiva was found in the TTd/wBP groups (*P<0.005* in comparison to the corresponding n.c.). Bivariate correlation analyses confirmed a strong correlation between the levels of TTd-specific IgG in the serum and the relative abundance of TTd-specific B cells in the draining lymph nodes (*P<0.01*; for BALB/c and C57BL/6 mice, the Pearson correlation coefficients were 0.787 and 0.832, respectively).

### The Greatest Proliferation of Draining SMLN Cells in Response to TTd was Observed using wBP as an Adjuvant

**Figure 5:**
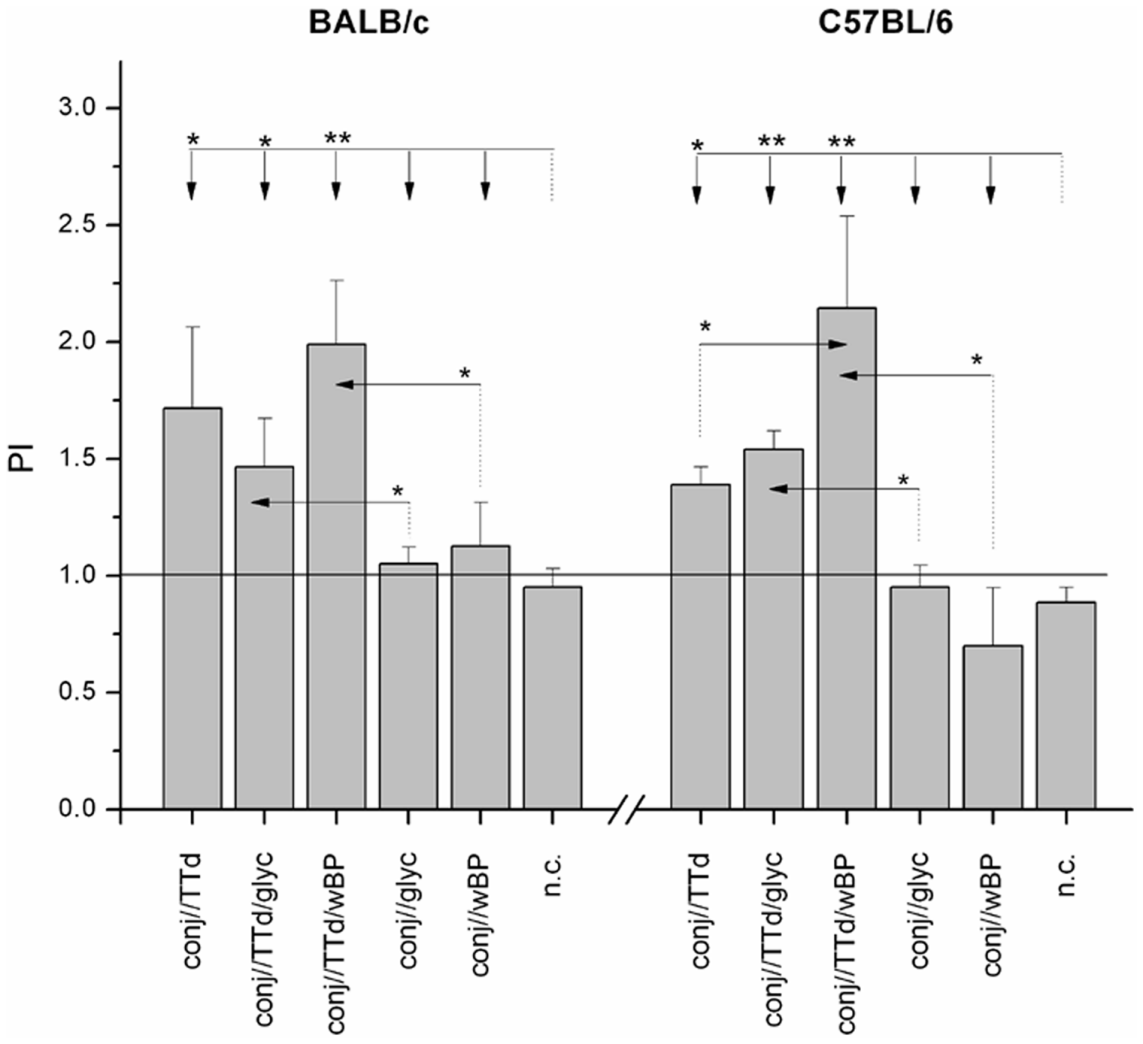
The proliferation indices (PI) of TTd-stimulated SMLN cells from BALB/c and C57BL/6 mice immunized via the conjunctiva according to the assigned immunization protocol. The numbers of viable SMLN cells were assessed by MTT assay following a 48 h cultivation in 10% FCS/50 µM β-mercaptoethanol/RPMI 1640 medium supplemented or not with TTd (5 µg/ml). PIs were calculated for each mouse. The results are presented as the mean PI ± SE for each experimental group (n = 10). The statistical significance of the differences in PIs between groups treated according to the assigned protocols was determined by *t*-test (*P<*0.05*, *P<*0.005**). The reference group is indicated by a dotted line, and the comparison group is indicated by an arrow.

SMLN cells from immunized mice were used to perform a cell proliferation assay (Fig. 5). Following immunization via the conjunctiva, both mouse strains showed almost identical trends in proliferation index values following stimulation with TTd. Among the groups that were immunized via the conjunctiva, SMLN cells from the TTd/wBP group showed the highest proliferation index values in response to TTd. In C57BL/6 mice, PIs recorded for the conj//TTd/wBP group were significantly higher than those for the conj//TTd group.

### Immunization with TTd/wBP via CALT Promoted the Establishment of a Th1 TTd-specific Immune Response

**Figure 6:**
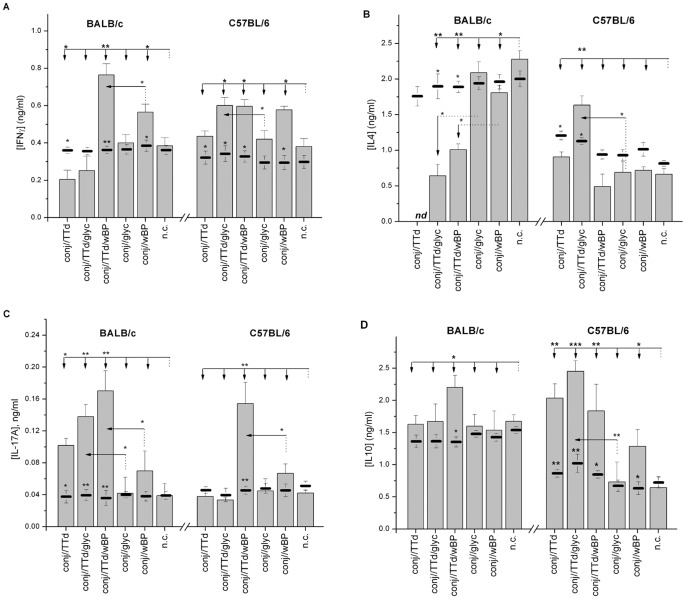
Levels of IFNγ (A), IL-4 (B), IL-17A (C) and IL-10 (D) in the supernatants from *in vitro* TTd-stimulated SMLN cells obtained from age-matched control mice (n.c.) and mice immunized via the conjunctiva according to the assigned protocol (bars). SMLN cells were cultivated at 37°C in 5% CO_2_ for 48 h in 10% FCS/RPMI 1640/50 µM β-mercaptoethanol supplemented with 5 µg/ml TTd. The levels of cytokines in the supernatants of corresponding SMLN cells incubated in 10% FCS/RPMI 1640/50 µM β-mercaptoethanol under similar conditions (non-stimulated cells) are indicated by solid lines. The results are presented as the mean concentration ± SE (n = 10). Concentrations of cytokines in supernatants of TTd-stimulated cultures were compared by *t-*tests. The reference group is indicated by a dotted line, and the comparison group is indicated by an arrow. A *t*-test was also used for the comparison of cytokine concentration in supernatants of corresponding non-stimulated and TTd-stimulated cultures, and the statistical significance of the differences is marked next to the solid bar, indicating the level of the cytokine within the non-stimulated culture. The levels of statistical significance are assigned as follows: P<0.05*, P<0.005**, P<0.0005***_._

SMLN cells from immunized and non-immunized control BALB/c and C57BL/6 mice were stimulated *in vitro* with TTd and analyzed for the production of IFNγ (a marker of Th1 responses), IL-4 (a marker of Th2 responses), IL-17A (a marker of Th17 responses) and IL-10 (a cytokine with regulatory and anti-inflammatory roles) (Fig. 6). As shown in Fig. 6, SMLN cells produced the tested cytokines without any additional stimulation during *in vitro* cultivation. Some treatment-dependent differences in the production of specific cytokines were marked, although the majority of the differences were not significant (data not shown). However, *in vitro* TTd stimulation generally altered the production of the tested cytokines in the SMLN cultures derived from conjunctively immunized mice.

A conjunctival multi-dose application of TTd alone, without an adjuvant, in BALB/c mice resulted in the diminished capacity of draining SMLN cells to produce IFNγ (*P<0.*05 in comparison to the BALB/c n.c. group) and IL-4 (the concentration was below the limit of detection) but an enhanced capacity to produce IL-17A (*P<0.05* in comparison to the BALB/c n.c. group) following *in vitro* TTd stimulation. In C57BL/6 mice treated in an identical manner, TTd stimulation of SMLN cells induced IL-10 secretion (*P<0.005* in comparison with the C57BL/6 n.c. group), whereas the production of IFNγ, IL-17A and IL-4 was not affected in comparison to non-immunized mice.

Conj//TTd/glyc immunization caused a reduction in the capacity of SMLN cells from BALB/c mice to secrete IFNγ and IL-4 (*P<0.005* in comparison to the n.c. group) upon TTd stimulation. In these cultures, only the secretion of IL-17A was augmented (*P<0.005* in comparison to the n.c. group). In contrast, SMLN cells from conj//TTd/glyc-immunized C57BL/6 mice produced IFNγ (*P<0.05*) and IL-4 (*P<0.005*) in higher quantities upon *in vitro* TTd stimulation than did the SMLN cells from control mice, whereas the production of IL-17A was similar to that detected in SMLN cultures from non-treated mice.

Significantly higher levels of both IFNγ (*P<0.005* for BALB/c, *P<0.05* for C57BL/6) and IL-17A (*P<0.005* for both strains) upon *in vitro* TTd stimulation were detected in SMLN cell cultures obtained from conj//TTd/wBP-treated mice compared to the corresponding TTd-stimulated control cell cultures obtained from non-immunized mice. The increased production of IFNγ upon TTd stimulation was also observed in cultures from conj//wBP-immunized mice (for both mouse strains, *P<0.05* in comparison to the corresponding n.c. group), whereas the production of IL-17A was not significantly greater than that observed in corresponding non-stimulated cultures. In TTd-stimulated cultures of SMLN cells from conj//TTd/wBP BALB/c mice, IL-10 production was also stimulated (*P<0.05*) in addition to in IFNγ and IL-17A production, whereas IL-4 production was diminished (*P<0.005*) in comparison to a reference culture of non-treated murine lymph node cells. In C57BL/6 mice, TTd stimulation did not affect the secretion of IL-4 by cells from conj//TTd/wBP lymph nodes but augmented the production of IL-10 (*P<0.005* in comparison to the n.c. group).

### Conjunctival Immunization with TTd Mixed with wBP Partially Protects BALB/c and C57BL/6 Mice Against Lethal Challenge with TTn

**Figure 7:**
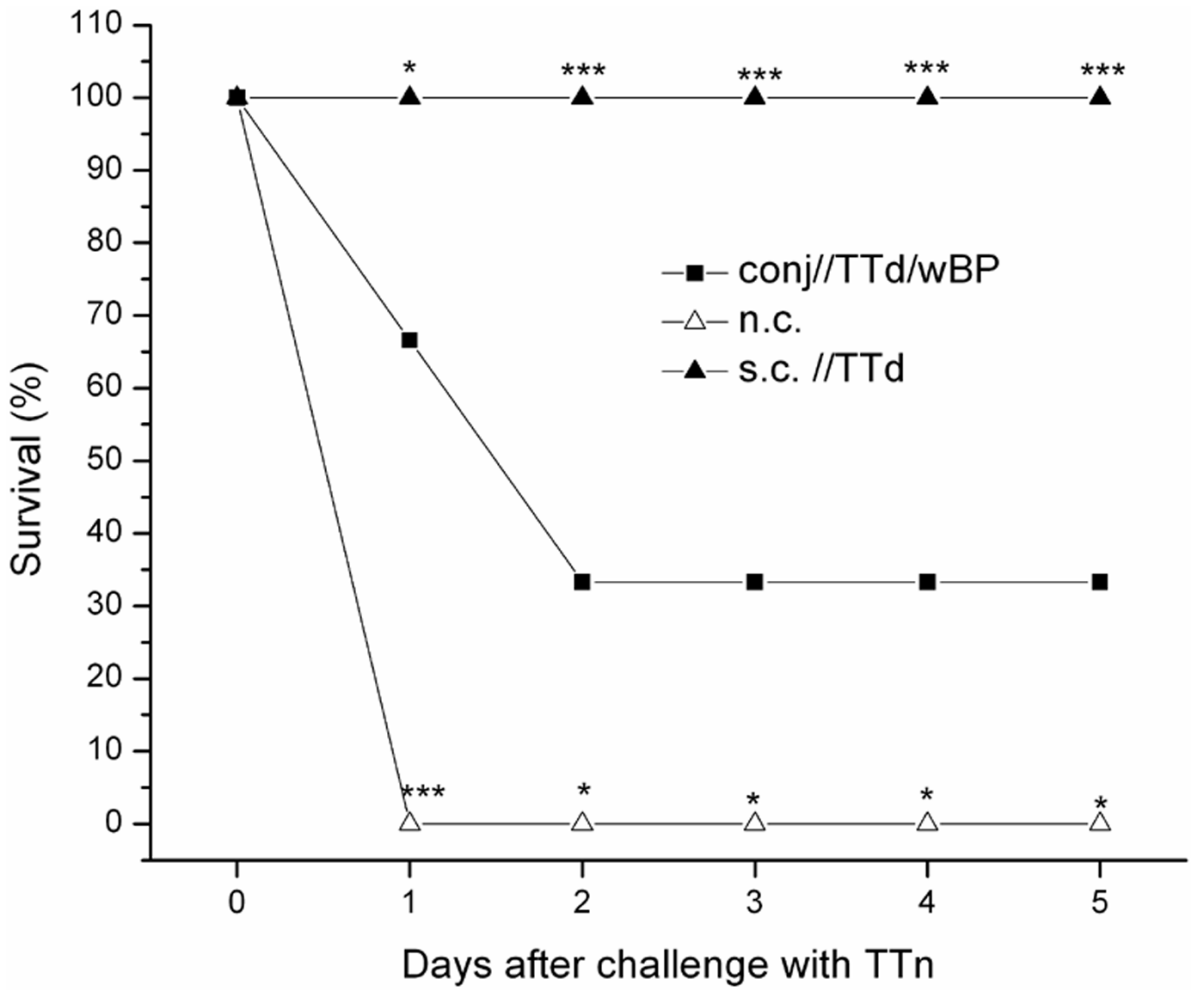
Survival rate of BALB/c and C57BL/6 mice immunized with TTd subcutaneously (s.c.//TTd), TTd mixed with wBP via the conjunctiva (conj//TTd/wBP) and non-treated age-matched mice (n.c.) upon challenge with tetanus toxin (TTn). Mice were immunized on days 0, 7 and 14 (100 µg TTd per dose) and four weeks after the completion of the specified immunization protocol, mice were challenged by i.p. administration of 2×LD_50_ of TTn. In both strains, survival rates were the same; hence, a representative plot is provided. Survival rates were monitored daily. The statistical significance of the differences in survival rates between conj//TTd/wBP (reference group) and s.c.//TTd or n.c. groups was determined by *t*-test (P<0.05*, P<0.005**_,_ P<0.0005***). The results are representative of two independent experiments, each comprising 6 mice per group.

Treatment-dependent survival upon challenge with a lethal dose of TTn was similar in both mouse strains. All subcutaneously immunized mice survived challenge with a lethal dose of TTn. All non-immunized mice, as well as mice conjunctively immunized with TTd alone, TTd/glyc, glyc or wBP, died on the first day post-challenge. Among the conjunctively immunized mice of both strains, only 33.3% of mice immunized with TTd/wBP survived the TTn challenge. It is evident that the survival rate of conj//TTd/wBP-immunized mice was lower than that of subcutaneously immunized mice (*P = 0.0001*) but was significantly greater than that of the non-treated age-matched control group and other groups treated via the conjunctiva (*P = 0.028*). The survival of sc//TTd- and conj//TTd/wBP-immunized mice, as well as age-matched control mice, upon challenge with a lethal dose of TTn is depicted in Fig. 7. Bivariate correlation analyses showed that the survival rates of both mouse strains upon challenge with TTn positively correlate to the level of sera anti-TTd IgG (*P<0.01*; for BALB/c and C57BL/6 mice, Pearson correlation coefficients were 0.817 and 0.511, respectively).

### No Ocular Irritation or Pathology was Observed at the Ocular Surface of Conjunctively Immunized Mice

Non-biased observers did not detect any signs of inflammation at the ocular surfaces of either mouse strain following immunization via the conjunctiva, as the eyes of non-immunized and sc//−immunized mice were visually identical to those of mice immunized via the conjunctiva. In addition, all mice remained healthy until the time of sacrifice, as there were no observed behavioral changes or weight loss during the course of immunization.

## Discussion

The delivery of antigens through mucosal surfaces is of remarkable interest because this route (i) mimics bacterial infection patterns and (ii) generates immunity at the major port of entry for such microorganisms [23]. Conjunctival immunization is an alternative approach to needle-based vaccine delivery against both mucosal (in particular, ocular) and systemic diseases, and more extensive research is needed to fully characterize the nature of the immune response stimulated by this route. It is likely that the response will vary for different antigens and will depend on whether adjuvants are required to enhance the specific response.

In this study, we evaluated characteristics of the conjunctival mucosal immunization route in profiling and comparing the local and systemic immune responses to the subcutaneous route using TTd as a model antigen. Here, we showed that immunization with TTd via the conjunctiva induced TTd-specific SIgA immune responses at the ocular surfaces in addition to the production of specific IgG and IgA antibodies detected in the serum. In both mouse strains, the SIgA concentrations in tears were higher in mice that were immunized via the conjunctiva than in mice that were sc//immunized, which is in agreement with previous publications using live attenuated vaccines [24], [25], [26]. The induction of B cell responses by conjunctival immunization was further supported by the presence of large numbers of mIgG^+^ TTd-specific cells within the SMLN total B cell population in all experimental groups. These data revealed that the levels of TTd-specific antibodies in murine sera induced by conjunctival administration of TTd strongly correlated with the presence of TTd-specific B cells in the draining lymph nodes. In all immunized groups of mice, the levels of TTd-specific antibodies in both tears and serum were higher in BALB/c mice than in C57BL/6 mice, which may be explained by the different genetic backgrounds of these mice.^C57BL/6and^ BALB/c^ mice are^ examples of mouse strains with predominantly Th-1 and Th2-type immune responses, respectively, both *in vivo* and *in vitro*. It is generally accepted that differences in the responses to pathogens by these two strains result from differences in the capacities for Th cell polarization, as BALB/c mice are genetically predisposed to establish a dominant Th2 immune response that is characterized, among other phenotypes, by cytokine production that promotes a humoral immune response.

As the most important finding, we demonstrated that the route of immunization highly influenced differentiation toward a Th1 or Th2 immune response. It is well known that subcutaneously administered TTd induces a Th2 immune response [27], [28], which is necessary for protection against tetanus. An analysis of cytokine secretion in draining SMLN cells upon *in vitro* stimulation with TTd revealed that immunization with TTd via the conjunctiva induced the skewing of TTd-specific immune responses toward a Th1-type response in both mouse strains, irrespective of the accompanying adjuvant, as determined by the stimulation of IFNγ secretion and/or diminished IL-4 production. Furthermore, this finding was supported by the prominent secretion of IFNγ-dependent antibody subclasses (IgG2a and IgG2c in BALB/c and C57BL/6 mice, respectively), which also indicate Th1-type immunity.

It is well known that an adjuvant is vital in inducing a strong immune response to the co-administered subunit antigen. Our use of wBP as an adjuvant was the best promoter of protective TTd-specific antibody secretion and profoundly modulated cytokine secretion. The characteristics of the immune responses observed in both mice strains upon immunization via the conjunctiva with wBP-adjuvanted TTd supported previous data showing that Th2 tetanus-specific immune responses are modified to a mixed Th1/Th2 response when TTd is co-injected with wBP cells [29]; thus, the whole-cell *B. pertussis* vaccine has the potential to suppress Th2-associated humoral responses to antigens if the antigen and adjuvant are administered simultaneously [30], and inactivated *B. pertussis* can promote the differentiation of Th1, as well as Th17, cells [31], [32]. Independent of the mouse strain used, conj//TTd/wBP immunization initiated mixed Th1/Th17 TTd-specific immune responses. Furthermore, mixed Th1/Th17 responses were initiated only when wBP was used as an adjuvant; in BALB/c mice, the conjunctival application of TTd alone or TTd adjuvanted with glyc impaired IFNγ secretion, and Th17 differentiation was impaired in similarly treated C57BL/6 mice. The induction of mixed Th1/Th17 immune responses may be crucial for defenses against intracellular parasites, as IFNγ and IL-17 synergistically enhance the resolution of such infections [33].

The results obtained from our model system are in line with a growing body of data that collectively imply that proper stimulation through TLRs is of immense importance for the establishment of protective immune responses. Furthermore, increases in the production of the pro-inflammatory cytokines IFNγ and IL-17 in most groups was accompanied by enhanced IL-10 secretion, which was most likely produced to counterbalance the pro-inflammatory responses. In addition to its pivotal role in Th17 skewing, TLR4 signaling in innate immune cells activates innate IL-10 production in response to wBP [30]. Therefore, we believe that this occurrence may explain the observed increases in IL-10 production in mice of both strains when wBP was used as an adjuvant. We propose that in our system, IL-10 acted primarily as an anti-inflammatory agent to balance IFNγ and IL-17 secretion, thus exerting a beneficial effect. It is likely that wBP limited inflammatory pathology by promoting the induction of IL-10-secreting type 1 regulatory T cells.

Although our aim and immunization protocol were not primarily targeting systemic protection, as most ocular surface diseases are localized, we obtained partial systemic protection in both mouse strains upon challenge with a lethal dose of TTn. In our model system, we induced primarily a Th1/Th17 response following immunization via the conjunctiva, whereas Th2-type responses obtained by sc//immunization have been shown to be important for full protection against tetanus [34], namely, neutralizing tetanus-specific antibodies are key factors for protection against tetanus. The secretion of these antibodies is greater in a Th2-type immune response, although skewing of the immune response toward Th1 does not completely block the production of such antibodies; in this case, antibody secretion occurs but is not dominant in the overall immune response.

By varying the TTd concentration, immunization scheme and design of delivery (e.g., not only simple mixing but also some coupling) in our model system, enhanced systemic protection against TTn in mice immunized via the conjunctiva will surely be achievable. However, the aim of this study was not to obtain a vaccine against TTn that could be applied via the conjunctiva but to use TTd as a model antigen to compare the immune profile of conjunctival immunization to a well-known and characterized immunization protocol to discern the features of conjunctival immunization and to determine whether this route of immunization could be used to protect against ocular infections.

Most vaccines are licensed for parenteral administration and fail to elicit appropriate mucosal immunity needed for protection against microorganisms that utilize mucosal ports of entry [35]. Consequently, effective conjunctival mucosal vaccines are needed, as they have the potential to dramatically contribute to the improvement of global eye health by stimulating protective immune responses at the site of infection.

We showed that the profile of the immune responses induced by the conjunctival mucosal route confers to the aforementioned advantages over parenteral vaccination. First, the most effective means of inducing an immune response at a specific effectors’ site is localized stimulation or, at the least, stimulation at a related site in terms of lymph drainage. Second, local exposure to an antigen resulted in much higher levels of specific SIgA in the region of exposure. Third, in addition to IgA responses, conjunctival vaccination induced systemic IgG responses, which represent a further defense against invasion by microorganisms or their products. Fourth, in addition to serum IgG and mucosal IgA antibodies, mucosal immunization stimulated cell-mediated responses, the latter being important in combating intracellular pathogens. In addition, conjunctival vaccination can also be safer and easier to dispense than traditional (parenteral) vaccines.

These results extend and support those of other authors showing that immunization via mucosal surfaces offers advantages over systemic delivery routes in protecting against intracellular pathogens. As conjunctival vaccines will be suitable for use in areas without appropriate health infrastructures, such vaccines have the potential to be successfully implemented in developing areas where access to medical personnel is limited and cooling chains are difficult to maintain. Therefore, the prevention of sight-threatening diseases might have a socioeconomic impact not only on a European level but also on a global level.
